# Bellmunt risk score enables survival prediction in men with metastatic castration resistant prostate cancer (mCRPC) undergoing PSMA-targeted radioligand therapy (LUMEN)

**DOI:** 10.1007/s00259-025-07492-9

**Published:** 2025-08-02

**Authors:** Thomas Büttner, Milka Marinova, Florian C. Gaertner, Barbara Kreppel, Leander Fritsche, Christian Immanuel, Gerd Geraths, Stefan Kürpig, Jim Küppers, Ingo G.H. Schmidt-Wolf, Markus Essler, Manuel Ritter, Philipp Krausewitz

**Affiliations:** 1 Department of Urology and Pediatric Urology, University Hospital Bonn, Bonn, Germany; 2 Department of Nuclear Medicine, University Hospital Bonn, Venusberg Campus 1, 53127 Bonn, Germany; 3 Department of Integrated Oncology, Center for Integrated Oncology (CIO), University Hospital Bonn, Bonn, Germany

**Keywords:** mCRPC, Lutetium, PSMA, Bellmunt Risk Score, Prediction, Survival

## Abstract

**Purpose:**

[^177^Lu]Lu-PSMA-617 therapy has emerged as a promising radioligand treatment for metastatic castration-resistant prostate cancer (mCRPC). Given the overall poor prognosis of mCRPC, accurately predicting the key endpoint overall survival (OS) remains an ongoing challenge, crucial for optimizing risk/benefit assessment prior to treatment. This study investigates the prognostic utility of the simple designed Bellmunt Risk Score (BRS) in mCRPC patients treated with [^177^Lu]Lu-PSMA-617.

**Methods:**

We retrospectively evaluated data from 386 mCRPC patients who had received [^177^Lu]Lu-PSMA-617 therapy at our center. BRS was constructed by attributing one point for each of the following risk factors: ECOG performance status (ECOG PS) > 0, hemoglobin < 10 g/dL, and the presence of liver metastases. Additionally, an enhanced version of the score including C-reactive protein (CRP) > 30 mg/dL and a modified version utilizing a semi-quantitative ECOG PS were developed. Statistical analysis included Cox regression, Kaplan-Meier estimates, concordance indices and time-dependent area under the curve (tAUC).

**Results:**

The BRS served as an independent predictor of OS in multivariable analysis. The estimated median OS was 17.6, 10.9, 6.6, and 2.7 months for patients with scores of 0, 1, 2 and 3, respectively (Log-Rank *P* < 0.001). The tAUC values for predicting 1-year and 2-year OS were 71.6% and 74.4%, respectively. The CRP-enhanced score further improved prognostic accuracy, achieving tAUCs of 75.7% for 1-year OS and 78.1% for 2-year OS.

**Conclusion:**

The Bellmunt Risk Score effectively stratifies mCRPC patients undergoing [^177^Lu]Lu-PSMA-617 therapy. Its straightforward design, based on readily accessible clinical parameters, ensures practicality and utility in real-world clinical settings.

**Supplementary Information:**

The online version contains supplementary material available at 10.1007/s00259-025-07492-9.

## Introduction

Despite recent advances in uro-oncology, the prognosis for patients with pre-treated metastatic castration-resistant prostate cancer (mCRPC) remains poor, with a median overall survival (OS) of approximately 11 to 15 months [1–3]. Lutetium-177 vipivotide tetraxetan prostate-specific membrane antigen (PSMA) therapy [^177^Lu]Lu-PSMA-617) represents a promising approach in the treatment of mCRPC. [^177^Lu]Lu-PSMA-617 is a PSMA-targeted radionuclide, which received European Medical Agency (EMA) approval in 2022 for mCRPC patients who have progressed following chemotherapy and androgen receptor signaling inhibitors (ARSI) [4]. The pivotal VISION trial demonstrated a significant OS benefit with a favorable safety profile [1]. Of note, OS remains the most critical endpoint, as patients undergoing [^177^Lu]Lu-PSMA-617 therapy have already received multiple lines of therapy and must carefully balance the potential benefits of further treatment against the associated burdens.

Reflecting the heterogeneity of mCRPC, survival benefits from [^177^Lu]Lu-PSMA-617 therapy vary significantly among patients, necessitating personalized prediction approaches. Several strategies have been developed, including laboratory findings, radiomics, circulating tumor DNA, and genomic markers [5–10]. To integrate these diverse risk factors, nomograms combining imaging variables with clinical and laboratory parameters have demonstrated significant prognostic value [11, 12]. However, in a real-world treatment setting, these variables are often unavailable, and the complexity of inputs may limit their practical application. Therefore, simple and handy risk scoring systems that enable rapid prognostic estimation remain an unmet need.

The Bellmunt Risk Score (BRS), initially developed for metastatic urothelial carcinoma (mUC), combines three risk factors: impaired general condition (Eastern Cooperative Oncology Group performance status [ECOG PS] > 0), reduced hemoglobin levels (< 10 g/dL), and the presence of liver metastases [13]. These factors are well-established prognostic markers across various tumor entities, including mCRPC [14–16]. Notably, all three parameters are standard clinical measures and are typically available in cohorts undergoing [^177^Lu]Lu-PSMA-617 therapy, making the BRS easily calculable at baseline. In a previous study, we demonstrated that the BRS is an effective predictor of OS in first-line treatment of mCRPC [17]. Furthermore, we identified potential improvements to this reliable risk score by incorporating ECOG PS as a semi-quantitative variable [17]. An enhancement to the BRS has been proposed in a metastatic urothelial cancer (mUC) cohort, integrating C-reactive protein (CRP) as an additional risk factor [18]. This modification is based on the understanding that cancer-related inflammation is closely linked to reduced treatment efficacy and poorer survival outcomes. CRP, as an acute-phase reactant, is an established surrogate marker for systemic inflammation, a critical prognostic factor [19]. Accordingly, CRP-enhanced BRS improved predictive accuracy in mUC patients [18].

The LUMEN trial (German Clinical Trial Registry: DRKS00035087) aimed to identify prognostic factors influencing outcomes of [^177^Lu]Lu-PSMA-617 therapy in mCRPC patients, within the context of a high-volume treatment center. In this analysis, we evaluated the Bellmunt Risk Score, along with its enhanced and modified versions, for their potential utility in predicting survival.

## Methods

### Patient cohort and ethics

**Fig. 1:**
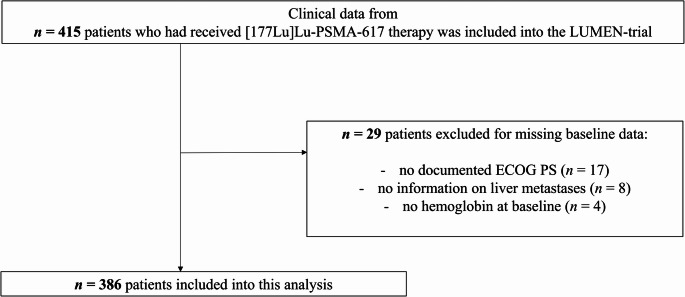
Cohort selection process

All patients who had received at least one cycle [^177^Lu]Lu-PSMA-617 therapy in the Department of Nuclear Medicine, University Hospital Bonn, from 2014 until December 2023 after an interdisciplinary decision as compassionate use or clinical routine were retrospectively included into the LUMEN cohort. All patients had PSMA-positive mCRPC, defined as at least one PSMA-positive metastatic lesion and no PSMA-negative lesions according to the VISION inclusion criteria [1]. The LUMEN trial assessed baseline variables, imaging and survival data. This subanalysis included only patients with complete baseline data for ECOG PS, hemoglobin, the presence of liver metastases, and CRP (Fig. 1). This study was approved by the local ethics committee (University Bonn, vote #2024-19-BO) and conducted in accordance with the Declaration of Helsinki.

### Baseline data

Baseline data of patients included demographics (e.g., age), medical history of mCRPC pretreatment (e.g., date of initial diagnosis, histology, duration, and type of prior therapies) alongside clinical and laboratory findings (ECOG PS, Body-Mass-Index [BMI], hemoglobin, alkaline phosphatase [ALP], lactate dehydrogenase [LDH], prostate-specific antigen [PSA], CRP, creatinine, and albumin). Additionally, the sites and number of metastases were documented based on baseline imaging.

### Radioligand therapy

PSMA-ligand (PSMA-617) was obtained from ABX GmbH (Radeberg, Germany). The preparation of [^177^Lu]Lu-PSMA-617 has been explained in detail in a previous publication [20]. The treatment solution was administered as bolus followed by 1000 ml of crystalloid fluid. All patients were discharged 48 h after therapy according to the rules of the national regulation “Richtline Strahlenschutz in der Medizin”. Treatment was continued until progression or intolerable toxicity.

### Bellmunt Risk Score (BRS)

The BRS was determined by assigning one point for each of the following risk factors: ECOG PS > 0, hemoglobin < 10 g/dL, and the presence of liver metastases, resulting in a total score ranging from 0 to 3.

### Modified Bellmunt Risk Score

The BRS was modified by replacing the traditional ECOG PS cut-off (> 0) with a semi-quantitative numeric value. One point was still assigned for hemoglobin levels < 10 g/dL and for the presence of liver metastases, resulting in a total score ranging from 0 to 7 [17].

### Enhanced Bellmunt Risk Score

The BRS was further refined by incorporating an additional risk factor: CRP > 30 mg/L, which was assigned one point. As previously described, this modification results in a total score ranging from 0 to 4 [18].

### Endpoints

Overall survival (OS) was defined as the time from the first cycle of [^177^Lu]Lu-PSMA-617 to death from any cause (event) or the date of last follow-up (censored). Time-dependent area under the curve (tAUC) and the concordance index were calculated to assess the longitudinal predictive accuracy of the score for OS.

### Statistics

Baseline data were summarized as means ± standard deviation (SD) and medians with interquartile range (IQR). Survival hazards were estimated using univariate and multivariate Cox regression analyses. Any continuous independent variables were converted into factors based on maximally selected rank statistics and clinical reasoning (e.g., PSA cut-off at 70 ng/mL). Only variables with significant hazards in univariate analysis were included in the multivariate models. In addition, the individual risk factors were excluded from multivariate models when the composite BRS was included to avoid multicollinearity. Survival comparisons were performed using Kaplan-Meier estimators and Log-Rank tests. tAUCs and Harrell’s C-index were applied to assess the predictive performance of the risk scores over time. A *p*-value < 0.05 was considered statistically significant. All statistical analyses were performed in RStudio (v2024.09.0 + 375), using the packages listed in Supp. Table 3.

## Results

A total of 386 patients met the inclusion criteria. Key baseline characteristics are summarized in Table 1, with a full description provided in Supp. Table 1. Approximately one-third (31.6%) of patients had no risk factors, 38.3% had one risk factor, 25.4% had two, and 4.7% had three risk factors, corresponding to their BRS. Thus, the cohort was split into similarly sized groups: low-risk (0 points), intermediate-risk (1 point), and high-risk (2 + points) patients. Further characteristics of the cohort resemble a typical mCRPC population of advanced age (median: 72.8 years), elevated PSA levels (median: 158 ng/mL), and significant metastatic burden (> 80%, with extra-axial bone metastases and > 25% with visceral metastases). The estimated median OS for the overall cohort was 11.0 months (95% confidence interval [95% CI]: 9.2–12.2).

**Table 1:** Key baseline characteristics of mCRPC patients at the start of [^177^Lu]Lu-PSMA-617 therapy

Parameter	Overall *n* = 386
Age (years)	
Mean (SD)	71.9 (8.5)
Median [IQR]	72.8 [66.4–77.9]
ECOG PS	
0	169 (43.8%)
1	148 (38.3%)
2	61 (15.8%)
3	8 (2.1%)
Metastatic count	
< 5	9 (2.3%)
5–10	40 (10.4%)
> 10	315 (81.6%)
Unknown	22 (5.7%)
Selected Metastatic sites	
Bone– axial only	56 (14.5%)
Bone– including non-axial	313 (81.1%)
Visceral (overall)	99 (25.6%)
Liver	68 (17.6%)
PSA (ng/mL)	
Mean (SD)	419 (718)
Median [IQR]	158 [35.0–519]
Hemoglobin (g/dL)	
Mean (SD)	10.9 (1.9)
Median [IQR]	11.0 [9.7–12.3]
CRP (g/L)	
Mean (SD)	30.2 (46.4)
Median [IQR]	8.0 [1.8–40.1]
Bellmunt Risk Score	
0	122 (31.6%)
1	148 (38.3%)
2	98 (25.4%)
3	18 (4.7%)

CRP = C-reactive protein, ECOG PS = Eastern Cooperative Oncology Group Performance Status, IQR = interquartile range, PSA = prostate-specific antigen, SD = standard deviation

### Radioligand treatment

The total number of [^177^Lu]Lu-PSMA-617 cycles administered was 1464 with a median of 3 cycles for each individual patient [IQR: 2–4]. The median cumulative activity was 18.7 GBq [IQR: 12.8–26.1] for the individual case.

### Survival hazards and estimators

**Fig. 2:**
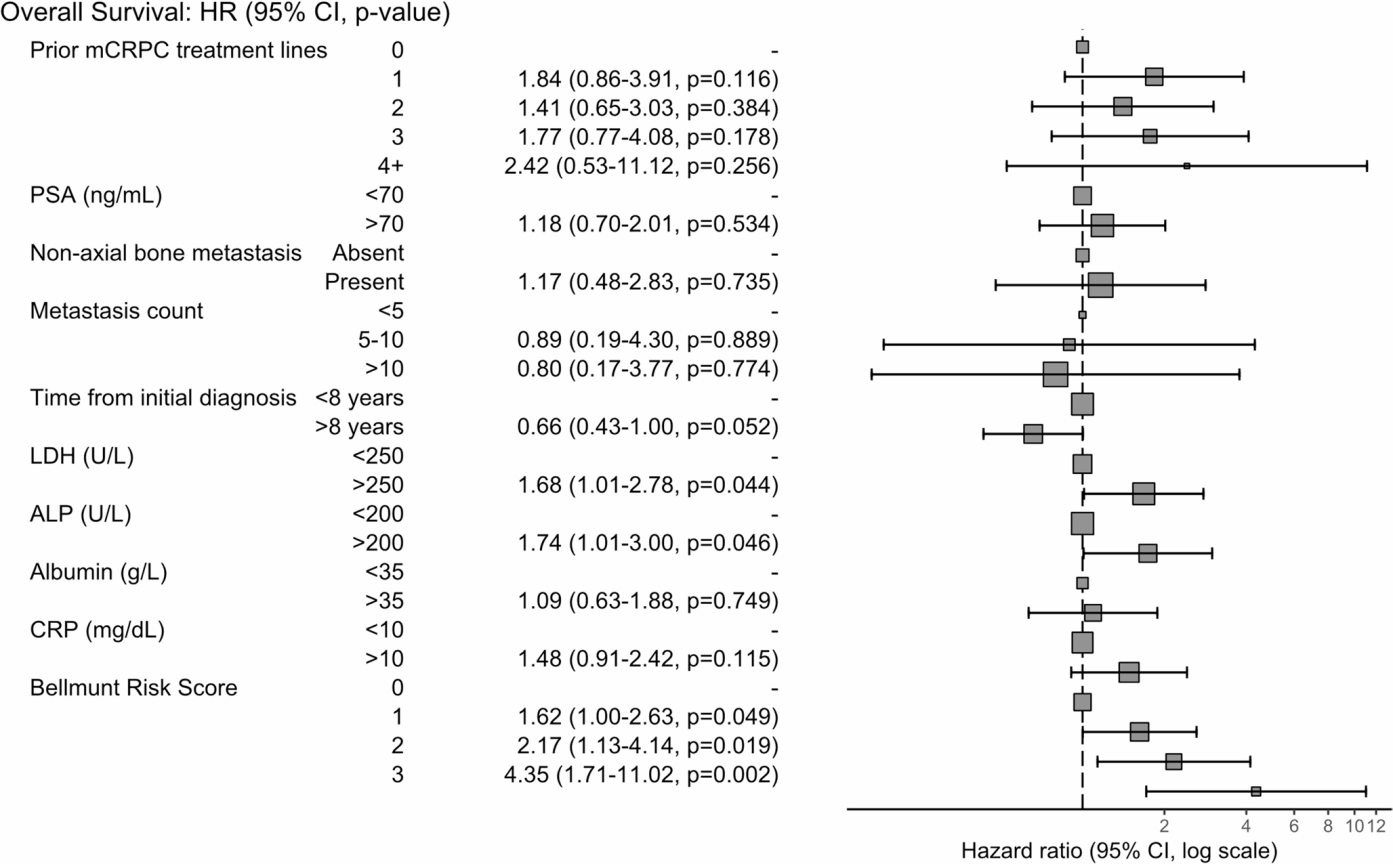
Multivariate Cox regression of risk factors for overall survival. HR = Hazard Ratio, 95% CI = 95% confidence interval

In univariate Cox regression, several variables were identified as significant predictors of OS. While age, BMI, and tumor grading did not show significant hazard ratios (HRs), ECOG PS, time from initial diagnosis, number of prior treatment lines for mCRPC, metastatic sites and count, and laboratory values including PSA, CRP, ALP, LDH, hemoglobin, and albumin were significantly associated with survival. Among these, the BRS demonstrated by far the strongest association, with the risk of death nearly doubling with each additional point of the score. The HR was 1.83 (95% CI: 1.41–2.38) for a score of 1, 3.63 (95% CI 2.69–4.90) for a score of 2, and 7.51 (95% CI 4.47–12.61) for a score of 3 (all *p* < 0.00). Results are summarized in Supp. Table 2. In multivariate Cox analysis, the BRS remained an independent survival predictor, alongside LDH and ALP, as depicted in Fig. 2.

**Fig. 3:**
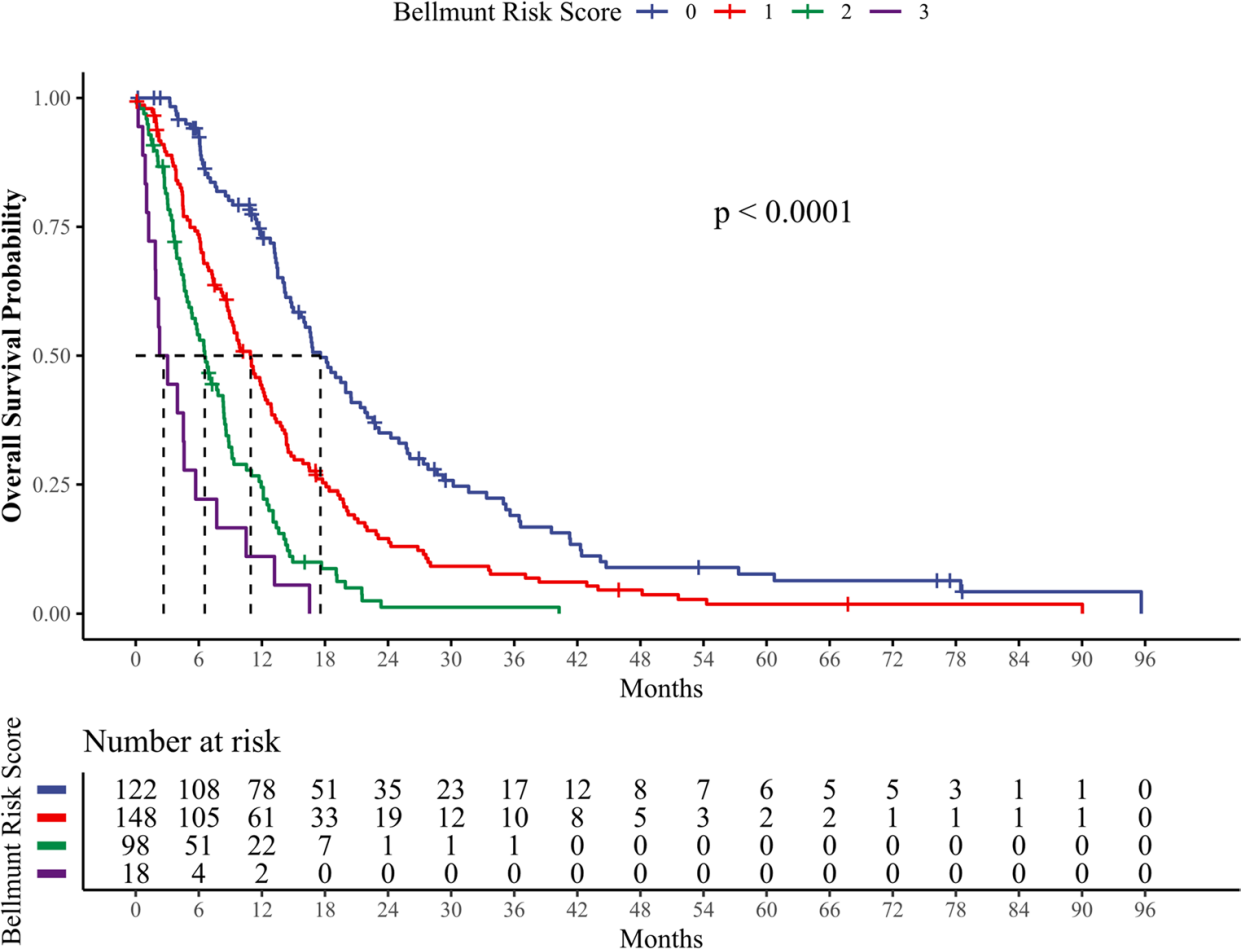
Kaplan-Meier estimators for Bellmunt Risk Score as a predictor of overall survival (OS). Estimated median OS was 18.1 months in patients with a score of 0, 9.9 months with a score of 1, 6.6 months for a score of 2 and 2.7 months for a score of 3 (Log-Rank *p* < 0.001)

In Kaplan-Meier estimators, the BRS effectively stratified patients into distinct groups based on survival outcomes. The estimated median OS was 17.6 months (95% CI 15.0–21.8) for patients with a score of 0, 10.9 months (95% CI: 8.9–12.5) for a score of 1, 6.6 months (95% CI: 5.3–8.4) for a score of 2, and 2.7 months (95% CI: 1.9–7.7) for a score of 3 (Log-Rank *p* < 0.001). The corresponding Kaplan-Meier plots are presented in Fig. 3. Six-months mortality rates were 6.6%, 25.7%, 44.9%, and 77.8% for patients with scores of 0, 1, 2, or 3, respectively, with a rate of 26.9% in the overall cohort. One-year mortality rates were 36.1%, 58.8%, 77.6%, and 88.9% for patients with respective scores of 0, 1, 2 or 3, compared to 57.8% in the overall cohort.

### Enhanced and modified Bellmunt Risk Score

**Fig. 4:**
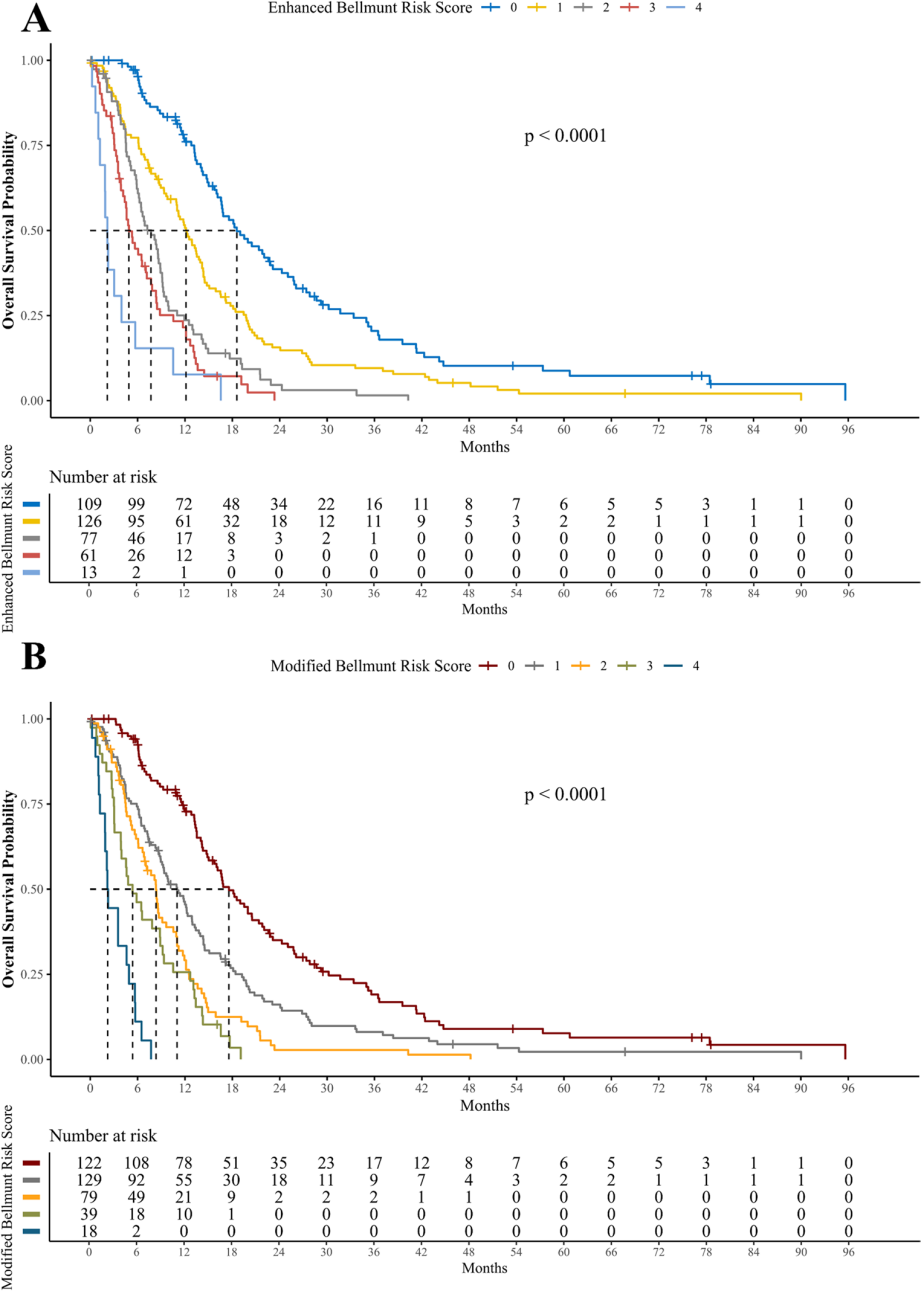
Kaplan-Meier estimators for enhanced (**A**) and modified (**B**) Bellmunt Risk Score as a predictor of overall survival (OS). Both scores were significantly associated with OS (Log-Rank *p* < 0.001 both)

Likewise the original score, both the enhanced and the modified versions of the BRS provided comparable risk group discrimination, as illustrated in Fig. 4. Notably, although the modified BRS potentially allows for scores up to 7, it ranged only from 0 to 4 in our cohort. When comparing the concordance indices x and tAUCs values for OS, both the modified (C-index: 0.67, 1-year tAUC: 72.1%) and enhanced BRS (C-index: 0.68, 1-year tAUC: 74.4%) showed improved predictive accuracy over the original score (C-index: 0.66, 1-year tAUC: 71.6%). While these improvements were relatively modest, they reached statistical significance for the enhanced BRS compared to the original score (*p* = 0.004 for C-index and *p* = 0.017 for 1-year survival tAUC). These findings are presented in Table 2 and Supp. Figure 1.

**Table 2:** Time-dependent area under the curve (tAUC) and concordance index (Harrell’s C) for original, enhanced and modified Bellmunt Risk Score as predictors of 6-months, 1-year, 2-year and 3-year overall survival (OS)

Time	tAUC (95% CI) for Overall Survival			
	Bellmunt Risk Score	Enhanced Bellmunt Risk Score	Modified Bellmunt Risk Score	*p*-value
6 months	74.3 (68.0–80.6)	77.6 (71.5–83.8)	75.4 (68.9–81.9)	**0.028**^**1**^
1 year	71.6 (65.4–77.7)	74.4 (68.3–70.6)	72.1 (65.9–78.4)	**0.017**^**1**^
2 years	75.7 (69.0–82.3)	78.1 (71.5–84.7)	76.4 (69.9–83.0)	n.s.
3 years	72.0 (62.6–81.3)	74.9 (66.4–83.4)	72.3 (62.9–81.8)	n.s.
Harrell’s C	0.66 (0.63–0.69)	0.68 (0.65–0.71)	0.67 (0.64–0.70)	**0.004**^**1**^

^1^for Bellmunt Risk Score vs. enhanced Bellmunt Risk Score, all other calculations were n.s.; 95% CI = 95% confidence interval; n.s. = not significant (*p* >0.05), tAUC = time-dependent Area Under the Curve

## Discussion

For the first time, our study demonstrated a strong and significant association between the Bellmunt Risk Score and Overall Survival, the most critical outcome for mCRPC patients undergoing [^177^Lu]Lu-PSMA-617 therapy. To our knowledge, this is the first investigation to establish this relationship, supported by data from a reasonably sized real-life cohort, further reinforcing the relevance of the BRS as a practical and robust prognostic tool in the context of metastatic prostate cancer management.

The primary strength of the BRS lies in its simplicity, as it relies exclusively on parameters that are widely accessible, even in real-world clinical settings. It incorporates patient characteristics such as ECOG PS, reflecting functional status, and hemoglobin levels, which serve as indicators of cachexia and physical decline [21, 22]. In addition, tumor-specific factors are represented, with liver metastases signaling tumor aggressiveness and hemoglobin levels potentially indicating bone marrow infiltration or bleeding [16, 22].

The calculation of the BRS is straightforward and memorable, requiring no computational support, which enhances its usability. Given that overall survival is a pivotal outcome measure in mCRPC, the BRS effectively stratifies patients into low, intermediate, or high-risk groups, facilitating translation into clinical practice and aiding in patient counseling.

Consequently, this study represents the first proposal of a simple, practical prognostic risk classification system for patients undergoing [^177^Lu]Lu-PSMA-617 therapy, contrasting with previously published studies that primarily focus on single risk factors or rely on complex and less accessible nomograms [5–10]. This simplicity makes the BRS particularly valuable for integration into routine care and decision-making.

To contextualize the BRS’s performance, we compared its predictive accuracy to a validated, international nomogram by Gafita et al., which integrates both clinical and PSMA-PET imaging variables [12]. Noteworthy, our cohorts exhibited comparable key baseline variables such as ECOG PS ≥ 2 proportions (17.9% vs. 12% in the development cohort and 23% in the validation cohort) or median PSA (158 ng/mL vs. 117 mg/mL in the development cohort and 138 ng/mL in the validation cohort) [12]. While we acknowledge the statistical nuances of comparing a simple, semi-quantitative score with a continuous nomogram, the BRS demonstrated a comparable prognostic performance, with a C-index of 0.66 versus 0.71 for the Gafita nomogram. Furthermore, when enhancing the BRS with CRP, the C-index rose to a more competitive 0.68, underscoring that a straightforward, clinically-based score can offer robust prognostic accuracy without requiring complex inputs [210]. However, the limitations of C-Index in censored survival data should be noted here, and the auspicious tAUCs may be a better indicator of BRS’ utility [23]. Due to a lack of published tAUCs in other prognostic models, a comparison cannot be performed though.

Notably, both the longitudinal accuracy of the BRS and the additional benefits of its modifications were lower in this study compared to our first-line mCRPC cohort [17]. Potential reasons for these discrepancies include substantial differences in treatment settings, cohort characteristics, and sample sizes. Despite these variations, the present study reaffirms the prognostic value of the BRS in mCRPC. With the distribution of key risk factors (hemoglobin, ECOG PS, and the presence of liver metastases) and median OS closely aligning with previous reports, our cohort appears representative of larger mCRPC populations scheduled for [^177^Lu]Lu-PSMA-617 therapy [24, 25].

Hence, our observations suggest several practical implications for clinical decision-making: for patients with a BRS of 0, a median survival of nearly 18 months can be anticipated. In this low-risk group, managing therapy-specific toxicities may take on greater importance, as these patients are likely to benefit from a longer treatment course.

In this context, it is worth noting that a heavy tumor burden observed in imaging or a high PSA should not discourage treatment in these men. In contrast, the subset of men with a BRS of 3 demonstrated a markedly poor median OS of just 2.7 months. For this high-risk subgroup, a thorough risk-benefit assessment is critical to determine the appropriateness for further treatment, along with intensified palliative measures. On the other hand, following the promising outcomes of earlier phase II trials, it is expected that the indications for [^177^Lu]Lu-PSMA-617 will expand in the future [26–28]. This anticipated expansion will likely increase the demand for [^177^Lu]Lu-PSMA-617 therapy slots, potentially leading to constraints in therapeutic capacity. In this context, the BRS could also serve as a valuable tool for prioritizing patient selection, ensuring that the [^177^Lu]Lu-PSMA-617 therapy is allocated effectively to those most likely to benefit.

### Limitations

Several limitations should be considered when interpreting these results. While our study encompasses the largest real-world [^177^Lu]Lu-PSMA-617 cohort described to date [25, 29], leveraging the strengths of a high-volume, high-experience center, potential biases remain due to its single-center design and retrospective nature. ECOG PS, as an essential part of the BRS, remains a subjective assessment of patients’ general condition. Inter-rater reliability is uncertain, with both reports of good and bad agreement among health-care professionals existing [30].

## Conclusion

The Bellmunt Risk Score serves as a significant and easily assessable prognostic tool for predicting survival in mCRPC patients undergoing [^177^Lu]Lu-PSMA-617 therapy. Its clinical utility lies in its robust ability to reliably estimate OS, the key outcome parameter, making it a valuable tool for personalized treatment stratification in this patient population. Furthermore, its simplicity and reliance on routinely available clinical parameters ensure its practical applicability in real-life daily clinical practice.

## Supplementary Information

Below is the link to the electronic supplementary material.

Supplementary file1 (DOCX 235 KB)

## Data Availability

The datasets generated analyzed during the current study are available from the corresponding author on reasonable request.
