# Supplementary material for: Bellmunt risk score enables survival prediction in men with metastatic castration resistant prostate cancer (mCRPC) undergoing PSMA-targeted radioligand therapy (LUMEN)
Source: Eur J Nucl Med Mol Imaging. 2025 Aug 2;53(2):931–9. doi: 10.1007/s00259-025-07492-9 (PMC12830477; doi:10.1007/s00259-025-07492-9)

**Supplementary Material**

Bellmunt Risk Score enables survival prediction in men with metastatic castration resistant prostate cancer undergoing PSMA-targeted radioligand therapy (LUMEN)

**Supp. Table 1** Full baseline characteristics of the cohort

| **Characteristic** | **Overall**  ***n* = 386** |
| --- | --- |
| Age (years) |  |
| Mean (SD) | 71.9 (8.5) |
| Median [IQR] | 72.8 [66.4 – 77.9] |
| Body-Mass-Index (kg/cm^2^) |  |
| Mean (SD) | 25.9 (4.2) |
| Median [IQR] | 25.4 [23.3 – 28.2] |
| Unknown | 11 (2.8%) |
| ECOG PS |  |
| 0 | 169 (43.8%) |
| 1 | 148 (38.3%) |
| 2 | 61 (15.8%) |
| 3 | 8 (2.1%) |
| Time from first diagnosis of prostate cancer (months) |  |
| Mean (SD) | 115.1 (204.3) |
| Median (Range) | 71.5 (37.8, 130.1) |
| Unknown | 8 (2.1%) |
| ISUP grading group |  |
| 1 | 16 (4.1%) |
| 2 | 69 (17.9%) |
| 3 | 64 (16.6%) |
| 4 | 118 (30.6%) |
| 5 | 27 (7.0%) |
| Unknown | 92 (23.8%) |
| Prior treatment lines for mCRPC |  |
| 0 | 44 (11.4%) |
| 1 | 126 (32.6%) |
| 2 | 140 (36.3%) |
| 3 | 64 (16.6%) |
| 4+ | 7 (1.8%) |
| Unknown | 5 (1.3%) |
| Metastatic count |  |
| <5 | 9 (2.3%) |
| 5-10 | 40 (10.4%) |
| >10 | 315 (81.6%) |
| Unknown | 22 (5.7%) |
| Selected Metastatic sites |  |
| Bone – axial only | 56 (14.5%) |
| Bone – including non-axial | 313 (81.1%) |
| Visceral | 99 (25.6%) |
| PSA (ng/mL) |  |
| Mean (SD) | 419 (718) |
| Median [IQR] | 158 [35.0 – 519] |
| Hemoglobin (g/dL) |  |
| Mean (SD) | 10.9 (1.9) |
| Median [IQR] | 11.0 [9.7 – 12.3] |
| Albumin (g/L) |  |
| Mean (SD) | 38.7 (5.2) |
| Median [IQR] | 38.8 [35.2 – 42.5] |
| Unknown | 236 (61.1%) |
| Creatinine (mg/dL) |  |
| Mean (SD) | 1.0 (0.7) |
| Median [IQR] | 0.9 [0.7 – 1.1] |
| ALP (U/L) |  |
| Mean (SD) | 255 (314) |
| Median [IQR] | 139 [77.0 – 305] |
| Unknown | 3 (0.8%) |
| LDH (U/L) |  |
| Mean (SD) | 431 (410) |
| Median [IQR] | 280 [219 – 444] |
| Unknown | 1 (0.3%) |
| CRP (g/L) |  |
| Mean (SD) | 30.2 (46.4) |
| Median [IQR] | 8.0 [1.8 – 40.1] |
| Bellmunt Risk Score |  |
| 0 | 122 (31.6%) |
| 1 | 148 (38.3%) |
| 2 | 98 (25.4%) |
| 3 | 18 (4.7%) |
| SD = standard deviation, IQR = interquartile range, ECOG PS = Eastern Cooperative Oncology Group Performance Status, mCRPC = metastatic castration-resistant prostate cancer, ISUP = International Society of Urological Pathology, PSA = Prostate-specific antigen, ALP = alkaline phosphatase, LDH = lactate dehydrogenase, CRP = C-reactive protein | |

**Supp. Table 2** Univariate Cox regression analysis of risk factors for Overall Survival (OS)

| **Variable** | **OS** | |
| --- | --- | --- |
|  | **HR (95% CI)** | ***P*** |
| Age >75 years | 0.89 (0.72-1.11) | 0.301 |
| BMI >30 | 0.88 (0.64-1.20) | 0.423 |
| ECOG PS |  |  |
| 0 | *Ref* |  |
| 1 | 1.56 (1.23-1.97) | **<0.001** |
| 2 | 2.91 (2.14-3.97) | **<0.001** |
| 3 | 4.98 (2.42-10.26) | **<0.001** |
| Time from first diagnosis > 8 years | 0.62 (0.49-0.77) | **<0.001** |
| ISUP grading group |  |  |
| 1 | *Ref* |  |
| 2 | 1.15 (0.64-2.07) | 0.638 |
| 3 | 1.24 (0.69-2.22) | 0.476 |
| 4 | 1.74 (1.00-3.05) | 0.052 |
| 5 | 1.57 (0.81-3.03) | 0.183 |
| Prior treatment lines for mCRPC |  |  |
| 0 | *Ref* |  |
| 1 | 2.06 (1.37-3.09) | **0.001** |
| 2 | 2.34 (1.56-3.49) | **<0.001** |
| 3 | 2.06 (1.33-3.21) | **0.001** |
| 4+ | 4.17 (1.90-9.16) | **0.001** |
| Metastatic count |  |  |
| <5 | *Ref* |  |
| 5-10 | 1.13 (0.40-3.22) | 0.813 |
| >10 | 2.85 (1.06-7.65) | **0.038** |
| Presence of non-axial bone metastases | 1.75 (1.27-2.42) | **0.001** |
| Presence of visceral metastases | 1.55 (1.22-1.97) | **<0.001** |
| PSA > 70 ng/mL | 1.85 (1.48-2.37) | **<0.001** |
| Hemoglobin > 10 g/dL | 0.37 (0.29-0.46) | **<0.001** |
| ALP > 200 U/L | 2.45 (1.95-3.08) | **<0.001** |
| LDH > 250 U/L) | 2.31 (1.84-2.90) | **<0.001** |
| Albumin > 35 g/L | 0.53 (0.36-0.79) | **0.002** |
| CRP > 30 g/L | 2.36 (1.90-2.94) | **<0.001** |
| Bellmunt Risk Score |  |  |
| 0 | *Ref* |  |
| 1 | 1.83 (1.41-2.38) | **<0.001** |
| 2 | 3.63 (2.69-4.90) | **<0.001** |
| 3 | 7.51 (4.47-12.61) | **<0.001** |
| HR = Hazard Ratio, 95% CI = 95% confidence interval, BMI = Body-Mass-Index, ECOG PS = Eastern Cooperative Oncology Group Performance Status, mCRPC = metastatic castration-resistant prostate cancer, ISUP = International Society of Urological Pathology, PSA = prostate-specific antigen, ALP = alkaline phosphatase, LDH = lactate dehydrogenase, CRP = C-reactive protein | | |

**Supp. Table 3** Packages imployed in RStudio v2024.09.0+375 for statistical analysis

| **Package** | **Version** | **DOI** |
| --- | --- | --- |
| survival | 3.7-0 | 10.32614/CRAN.package.survival |
| survminer | 0.4.9 | 10.32614/CRAN.package.survminer |
| rms | 6.8-2 | 10.32614/CRAN.package.rms |
| compareC | 1.2.3 | 10.32614/CRAN.package.compareC |
| TimeROC | 0.4 | 10.32614/CRAN.package.timeROC |
| finalfit | 1.0.8 | 10.32614/CRAN.package.finalfit |
| ggplot2 | 3.5.1 | 10.32614/CRAN.package.ggplot2 |
| ggpubr | 0.6.0 | 10.32614/CRAN.package.ggpubr |

**Supp. Figure 1** Time-dependent Area under the Curve (tAUC) calculated in monthly intervals (time t = months) for original, enhanced and modified Bellmunt Risk Score as predictors of Overall Survival. Dotted lines indicate 95 % confidence interval.


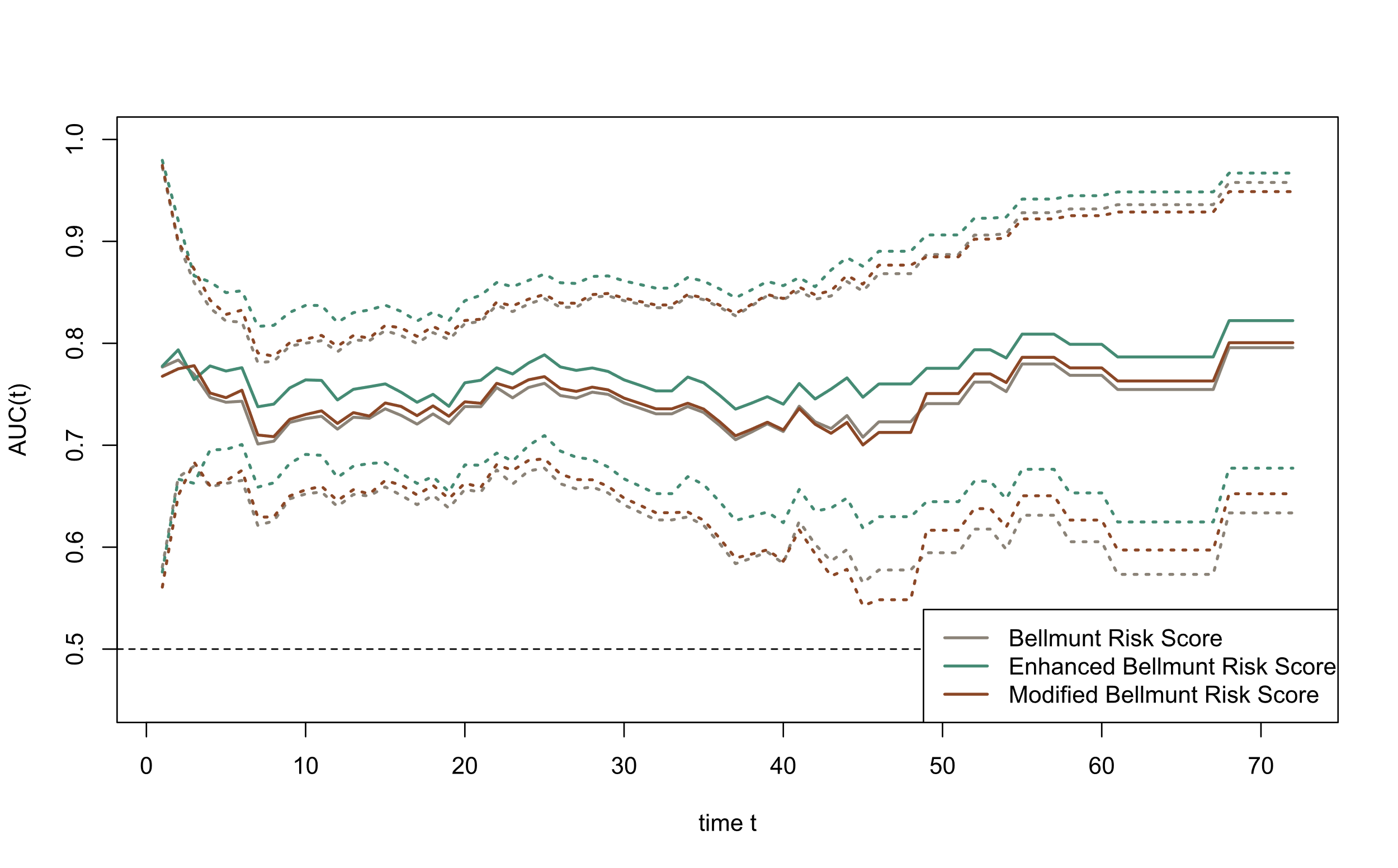

Supplement: Supplementary file 1 — Supplementary file1 (DOCX 235 KB) [file 259_2025_7492_MOESM1_ESM.docx]
